# On the Use of a Multimodal Optimizer for Fitting Neuron Models. Application to the Cerebellar Granule Cell

**DOI:** 10.3389/fninf.2021.663797

**Published:** 2021-06-03

**Authors:** Milagros Marín, Nicolás C. Cruz, Eva M. Ortigosa, María J. Sáez-Lara, Jesús A. Garrido, Richard R. Carrillo

**Affiliations:** ^1^Department of Biochemistry and Molecular Biology I, University of Granada, Granada, Spain; ^2^Department of Informatics, University of Almería, ceiA3, Almería, Spain; ^3^Department of Computer Architecture and Technology—CITIC, University of Granada, Granada, Spain

**Keywords:** granule cell, cerebellum, neuron model, optimization, adaptive exponential integrate-and-fire, multimodal evolutionary algorithm

## Abstract

This article extends a recent methodological workflow for creating realistic and computationally efficient neuron models whilst capturing essential aspects of single-neuron dynamics. We overcome the intrinsic limitations of the extant optimization methods by proposing an alternative optimization component based on multimodal algorithms. This approach can natively explore a diverse population of neuron model configurations. In contrast to methods that focus on a single global optimum, the multimodal method allows directly obtaining a set of promising solutions for a single but complex multi-feature objective function. The final sparse population of candidate solutions has to be analyzed and evaluated according to the biological plausibility and their objective to the target features by the expert. In order to illustrate the value of this approach, we base our proposal on the optimization of cerebellar granule cell (GrC) models that replicate the essential properties of the biological cell. Our results show the emerging variability of plausible sets of values that this type of neuron can adopt underlying complex spiking characteristics. Also, the set of selected cerebellar GrC models captured spiking dynamics closer to the reference model than the single model obtained with off-the-shelf parameter optimization algorithms used in our previous article. The method hereby proposed represents a valuable strategy for adjusting a varied population of realistic and simplified neuron models. It can be applied to other kinds of neuron models and biological contexts.

## Introduction

Large-scale neural network simulations composed of thousands or millions of neurons are useful for better understanding brain information processing primitives. Simplified single-neuron models of low computational cost and based on a few parameters have been proposed to reproduce neuronal firing patterns to encode and decode the information contained in electrophysiological recordings (Izhikevich, 2004; Shan et al., 2017; Marín et al., 2020). These models are required to meet efficiency and biological realism for hypothesizing the functional impact of relevant neuron properties within large-scale simulations. Since most simplified models (e.g., the integrate-and-fire neuron model family) contain abstract parameters that prevent direct adjustment with the biological counterpart biophysical features, optimization algorithms represent an attractive approach for precisely setting the parameters in this kind of neuron models (Druckmann et al., 2007; Jolivet et al., 2008; Friedrich et al., 2014; Pozzorini et al., 2015). However, accurately fitting their parameters to reproduce biological data can be considered a challenging optimization problem that still remains partially unsolved.

The cerebellum is a major center of the nervous system involved in fine motor control, somatosensory processing, and many other non-motor tasks (Schmahmann, 2019). One of the cerebellar neuron types, the granule cells (GrCs) are the most abundant neurons in the human brain (Lange, 1975; Williams and Herrup, 1988). The GrCs are thought to regulate the information transmission through the main afferent system of the cerebellum (Jörntell and Ekerot, 2006). Experimental recordings have characterized two of their main firing features, such as regular repetitive firing and latency to the first spike under injected step currents (D’Angelo et al., 1995, 1998, 2001; Masoli et al., 2017). Also, previous findings suggest intrinsic spiking resonance (as enhanced bursting activity during low-frequency sinusoidal current injections) preferentially in the theta-frequency band (around 5–12 Hz *in vitro* recordings of cerebellar GrCs in rodents; D’Angelo et al., 2001). This complex behavior has been proposed to strengthen the transmission of information in the cerebellar input layer (D’Angelo et al., 2001, 2009; Gandolfi et al., 2013). The definition of cerebellar GrC models that replicate these complex patterns represents an initial step towards understanding the functional role of resonance in information processing and the involvement of the GrCs in the synchronization and learning of the cerebellum.

The relevance of heterogeneity in the population of neurons of the same type with variances in their properties has been highlighted in computational experimentation (Lengler et al., 2013; Migliore et al., 2018). However, the benefits of high variance in terms of biodiversity of neurons in the signal processing of the brain remain largely unexplored. The variances in the neuron properties were demonstrated to enhance the speed, responsiveness and robustness of the spiking neuron networks. Thus, the intrinsic variability of neurons in the brain is proposed to crucially change the network dynamics and could have a role in information processing. The generation of heterogeneous populations of spiking neurons whose properties are closely matched with biological data is of utmost necessity as a first-step in the demonstration of this novel assumption.

As the complexity of neuron models and the available computational power have increased, the use of different optimization algorithms for tuning this kind of simple models has also grown (Van Geit et al., 2008). Consequently, there have been used optimizers for tuning the parameters of computationally efficient neuron models and reproducing certain biological behaviors in previous works. Some authors opt for algorithms with a solid mathematical component, such as the Sequential Quadratic Programming (SQP) method used to tune the modified generalized leaky integrate-and-fire (E-GLIF) model of a cerebellar Golgi cell (GoC; Geminiani et al., 2018). Other examples are the Downhill simplex method and L-BFGS-B, which are included in the open-source optimization framework “Optimizer” (Friedrich et al., 2014). However, the use of optimizers relying on randomness and nature-inspired principles with generic and minimal mathematical components (Lindfield and Penny, 2017) is also very popular among authors (Van Geit et al., 2008). For instance, the referred “Optimizer” framework offers Evolutionary algorithms (EAs) and Simulated Annealing too (Friedrich et al., 2014). The “BluePyOpt’ framework also relies on multi-objective EAs such as Non-dominated Sorting Genetic Algorithm-II (NSGA-II), Multi-Objective Covariance Matrix Adaptation Evolution Strategy (MO-CMA-ES), and IBEA (Van Geit et al., 2016). Similarly, Nair et al. (2015) fits the AdEx model of a cerebellar GrC using Particle Swarm Optimization (PSO), and Masoli et al. (2017) opts for the IBEA Genetic Algorithm (GA) to tune the detailed Hodgkin-Huxley (HH) model of a cerebellar GrC with the maximum ionic conductances.

Our previous work (Marín et al., 2020) proposed a tuning procedure based on traditional GAs (EAs based on basic genetic operators, such as crossover and mutation) for creating an adaptive exponential integrate-and-fire (AdEx) model of the cerebellar GrC. We proposed a complex objective function defined by the inherent properties mentioned above and measured as the accumulated distance between the *in vitro* recordings and the simulated responses of the neuron model that is being tuned. Finally, we selected and proposed a GrC model (a specific set of parameters of an AdEx generic neuron model) as the result of the process.

According to the previous literature review and independently of their class, the most used optimization strategies are either multi-objective (which by definition return a set of candidate solutions considering several criteria concurrently) or single-objective yet aimed at converging to a single optimal solution. However, the application of multimodal optimizers (Sareni and Krähenbühl, 1998; Jelasity et al., 2001; Shir et al., 2010) does not seem to be popular even though it has been found that it is possible to find real neurons and neuron models with very similar behavior but different parameters (Van Geit et al., 2008). Multi-Objective algorithms require working in parallel with different objective functions. They can find large sets (Pareto fronts) of equally valuable configurations at the expense of higher conceptual complexity than single-objective methods. On the contrary, standard single-objective methods work in a simpler background and aim at converging to a single solution, but this behavior can be problematic since evaluations rely on models and some of them might not be as valid as estimated. In this context, the use of a multimodal optimizer arises as a mid-term solution between the algorithms designed for converging to a single solution and those considering different objective functions and returning a set of equally valuable options for an expert to decide. More precisely, a multimodal algorithm will focus on a single objective function, but it will also identify different equivalent solutions that should be ultimately filtered by an expert on the neuron model. Moreover, taking into account potential problems such as noise in the experimental data, low model accuracy, and degenerate cases of the selected objective function, experts might prefer some promising solutions over those theoretically better (strictly in terms of the referred objective function).

The present workflow aims to overcome the intrinsic limitations of the current optimization approaches by identifying a sparse population of different optimal solutions in the search space for a single objective function integrating various features. In this regard, this article extends the methodology presented in Marín et al. (2020) proposing an alternative optimization component based on a multimodal EA for building realistic and computationally efficient neuron models. We base our proposal on the same complex parametric optimization problem as in Marín et al. (2020): optimizing cerebellar GrC models that replicate the essential firing properties of the biological cell, which are essentially the decrease of latency to the first spike and spike frequency increase when the injected step-current intensity is raised [intensity-frequency (I-F) curves], and spiking resonance at the theta-frequency band during sinusoidal current injections. The final population of candidate solutions has to be analyzed and evaluated according to the biological plausibility and their objective to the target features. In order to illustrate the value of this approach, we explore the resulting diversity of the population of cerebellar GrC models and their functional spiking dynamics. Our results show the variability of plausible sets of values that this type of neuron can adopt underlying these complex characteristics.

The rest of the article is structured as follows: “Methodological Workflow” section describes the methodological workflow proposed in this article. “Materials and Methods” section explains the neuron model whose parameters must be tuned, the corresponding optimization problem, and the multimodal optimizer. “Results” section presents the results achieved and the spiking dynamics simulated by the selected neuron configurations. Finally, “Discussion” section contains the conclusions and states some possible future work lines.

## Methodological Workflow

**Figure 1:**
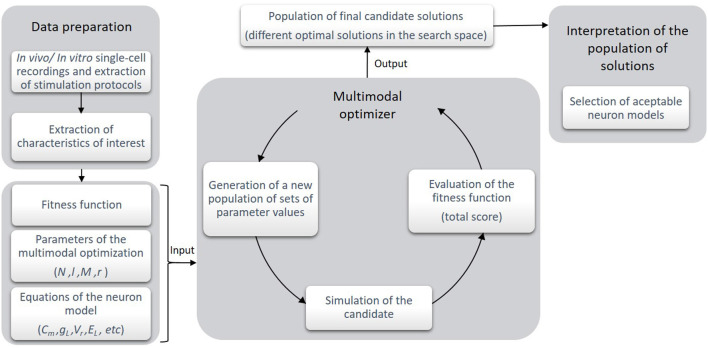
Stages of the optimization framework. At the first stage, the experimental recordings of the biological cells are obtained and the characteristics of interest are selected and calculated. Then, according to these data, a fitness (or objective) function is built and the parameters of the algorithm and the neuron model are selected. At the second stage, the execution of the multimodal optimizer takes place. The optimization process consists in generating a number of candidates (i.e., sets of parameter values) which are simulated and evaluated according to the objective function. Finally, as the output of this stage, a population of different candidate solutions is returned in a single execution of the algorithm. These selected candidates are illustrative of some local optima in the search space of the objective function. At this point, the expert interprets if the candidate solutions are as numerically valid as their scores estimate. Quantitatively and qualitatively, the best-ranked candidate solutions are selected as the neuron models proposed.

In this section, we present the structure of the proposed optimization workflow. Figure 1 briefly depicts the workflow chart of the methodology. The course of action runs as follows:

### Data Preparation

Firstly, the user has to select the particular firing properties of interest of the cell under study extracted from *in vitro* or *in vivo* electrophysiological recordings under specific stimulation protocols. These features (also named objectives) and their protocols define the target function that will drive the selection during the optimization procedure.

### Optimization Algorithm

The objective or fitness function, the optimization algorithm parameters and the neuron model parameters (all described in the sections below) correspond to the initial set-up of the optimization architecture. The multimodal algorithm performs an exploration of the parameter space using a simplified (point-neuron) model template. The neuron models with specific sets of parameters which are obtained during the search (also named candidate solutions) are evaluated according to the objective function. Those model configurations with the lowest total score and different enough from the rest are selected in each iteration and passed to the next optimization iterations during the optimization process. The output of this stage is a sparse population of candidate solutions that correspond to different sets of parameters that stand out in their zone of the search space.

### Selection and Interpretation of the Candidate Solutions

Once the algorithm has selected different promising model configurations, the user will validate the most suitable neurons among them. This selection runs in accordance with those neuron models which show biological plausibility of their parameters and reproduce with high realism the firing behavior of the real neuron.

## Materials and Methods

In order to demonstrate the potential of the optimization workflow, we applied this methodology to the case of the cerebellar granule cells (GrCs) as a proof-of-concept. This approach allows us to validate the sparse population of candidate solutions obtained and according to the features defined in the existing literature. This section starts by describing the computational neuron model, defining the optimization problem to solve and the experimental pieces of single-cell recordings. It ends with the technical details for simulation reproducibility and data analysis.

### Neuron Model Structure

Since GrCs have a compact electrotonic structure (Silver et al., 1992; D’Angelo et al., 1995; Delvendahl et al., 2015), single-compartment modeling is appropriate. One of the widely used computationally-efficient neuron models is the adaptive exponential integrate-and-fire (AdEx) model (Brette and Gerstner, 2005), but other types of point-neuron models could be considered in this first stage of the workflow (Figure 1). The AdEx model is capable of reproducing a diversity of neuronal dynamics customizing a few parameters (Naud et al., 2008). Its realism and great computational efficiency have been supported by several comparisons with detailed models and experimental recordings (Brette and Gerstner, 2005; Jolivet et al., 2008; Naud et al., 2008; Nair et al., 2015; Marín et al., 2020). Accurately fitting the model with respect to experimental measurements is not straightforward. The adaptation state variable of the AdEx model allows good fitness with different firing modes (e.g., regular discharge, bursting, delayed spiking, etc.) depending on specific parameters values (Jolivet et al., 2008; Naud et al., 2008). However, its nonlinearity makes the optimization of its parameters, challenging.

The AdEx model consists of only two coupled differential equations and a reset condition.

(1) $$C_{m}\frac{dv}{dt}=-g_{L}(V-E_{L})+g_{L}\Delta_{T}\exp\left(\frac{V-V_{T}}{\Delta_{T}}\right)$$

(2) $$\tau_{w}\frac{dw}{dt}=a\left(V-E_{L}\right)-w$$

(3) $$if\;V>V_{peak}\;then\;V\leftarrow V_{r}\;and\;w\leftarrow w+b$$

Equation (1) describes the evolution of the first state variable, namely membrane potential (*V*), during current injection (*I(t)*). Equation (2) describes the evolution of the second state variable, namely adaptation current (*w*). When the current *I(t)* drives *V* beyond the threshold potential (*V_T_*), then the exponential term of the slope factor (*Δ_T_*) in equation (1) dominates the action potential until *V* reaches the reset threshold potential (*V_peak_*). Then, the reset condition (3) determines that *V* is instantaneously set to *V_r_* and *w* is increased a fixed amount named *b*. Both equations (1) and (2) contain 10 free parameters that can be optimized in order to minimize an arbitrary objective function (namely the difference of the obtained neural model behavior with respect to a “desired behavior” for instance, reproducing firing characteristics of cell recordings). These parameters are: the total leak conductance (*g_L_*), the leak reversal potential (*E_L_*) and the membrane capacitance (*C_m_*) in equation (1) that model the passive membrane mechanisms; the parameters *Δ_T_* and *V_T_* in the exponential term of equation (1) model the spike generation and shape; the time constant parameter (*τ_w_*), the subthreshold adaptation (*a*) and the spike-triggered adaptation parameter (*b*) define the evolution of the state variable *w* in equation (2); the parameters *V_peak_* and *V_r_* that drive the reset condition as mentioned above. These parameters have been set within fixed ranges to constrain the exploring process (Table 1). The membrane potential was initially set to the same value as the leak reversal potential (*V_init_* = *E_L_*).

**Table 1:** Model parameter ranges established for the search space of the optimization process.

Parameters	Min. value	Max. value	Parameters	Min. value	Max. value
*C*_m_	0.1 pF	5.0 pF	*V*_T_	−60 mV	−20 mV
Δ_T_	1 mV	1,000 mV	*a*	−1 nS	1 nS
*E*_L_	−80 mV	−40 mV	*b*	−1 pA	1 pA
*V*_r_	−80 mV	−40 mV	*g*_L_	0.001 nS	10.0 nS
*V*_peak_	−20 mV	20 mV	τ_w_	1 ms	1,000 ms

### Model Context and Problem Definition

#### Selection of Features

In order to obtain a neuron model that replicates the behavior of the cerebellar GrCs we have selected some features which quantify some of the most characteristic firing properties of this neuron type: (1) mean frequency through repetitive firing discharge under direct current stimulation [equation (4)]; (2) latency to the first spike under direct current stimulation [equation (5)]; and (3) burst frequency in response to different sinusoidal current stimulation (stimulation with different oscillation frequencies) [equation (6)]. These features will be combined into a single objective or fitness function to be considered by the selected multimodal evolutionary optimizer.

#### Parameter Optimization

The parameter optimization is carried out minimizing the fitness function by weighting the difference of these quantified features with a reference taken from real electrophysiological recordings. Thus, the objective function is defined as the weighted sum of the scores of the specific features (*feature_score*) related to the spiking features, according to equations (4, 5 and 6). The definition of the objective function that contains all these features is extracted from Marín et al. (2020).

(4) $$feature\_score_{Mean\;frequency}=\sum_{i=1}^{n}\left[abs\left(MF_{simi}-MF_{\exp i}\right)\cdot w_{Mean\;frequency}\right]$$

(5) $$feature\_score_{First-spike\;latency}=\sum_{i=1}^{n}\left[abs\left(LA\;T_{simi}-LA\;T_{\exp i}\right)\cdot w_{First-spike\;latency}\right]$$

(6) $$feature\_score_{Burst\;frequency}=\sum_{i=1}^{n}\left[abs\left(BF_{simj}-BF_{\exp j}\right)\cdot w_{Burst\;frequency}\cdot \left(std(BF_{simj})+1\right)\right]$$

The feature score (score function of each feature or objective) is calculated as the absolute distance (*abs*) between the feature values extracted from the *in vitro* electrophysiological recordings (*exp*_i_) and the feature values extracted from the simulated traces from the neuron model (*sim*_i_). This is multiplied by the weight associated with each feature (*w*_Mean frequency_, *w*_First–spike latency_ and *w*_Burst frequency_). The weights of the burst frequency (*w*_Burst frequency_) and the mean frequency (*w*_Mean frequency_) were set to 1 as they both were measured in hertz (Hz) and show values in comparable scales. The weight of the first-spike latency (*w*_First–spike latency_) was weighted to 1,000 as it was measured in seconds (s). Hence, the algorithm equally weights 1 Hz-error at mean frequency feature, 1 ms-lag at first-spike latency and 1 Hz-error at burst frequency. However, the feature score can be modified (if enhancing the focus on a particular feature with respect to the others) or extended if some extra aspect is to be taken into consideration. For instance, a penalization was used in the definition of the burst frequency score (*feature*_score Burst frequency_) to assure the stability of bursts [as proposed in Marín et al. (2020); Equation (6)].

#### Feature Measurement

The experimental recordings of the repetitive discharge are the mean frequency (defined as the number of spikes divided by the stimulation time) during 1-s length step-current injections of 10, 16 and 22 pA. The latency to the first spike is defined as the time the neuron takes to elicit its first spike upon current stimulation. Both features are extracted from *in vitro* patch-clamp recordings performed from acute cerebellar slices of a population of cerebellar GrCs (Masoli et al., 2017). The spiking resonance in the theta-frequency range is a complex behavior determined by the burst frequency [as the inverse of the average inter-spike interval (ISI) of the output neuron] during each stimulation cycle. Then, the average burst frequencies are measured throughout 10 consecutive cycles of sinusoidal stimulation. As it occurred in the *in vitro* recordings, we have set the burst frequency to zero when one or no spike per cycle has been obtained in the simulated neurons. The stimulation protocol consists of sinusoidal current injections with 6-pA and 8-pA amplitudes, sustained by a 12-pA offset during 22.5 s. They generate spike bursts in correspondence with the positive phase of the stimulus (sinusoidal phase of 270°). These features are extracted from *in vitro* patch-clamp recordings performed from acute cerebellar slices of a single cerebellar GrC (D’Angelo et al., 2001). It is worth mentioning that since the number of cells differ in both reference sources (the former from a population of cells and the latter from a single cell), and for the sake of equality, we selected the mean feature value of the mean frequency and the first-spike latency as a target type of neuron. On the other hand, the reference data points for the resonance frequency used in the fitness function are based on a single neuron measurement.

### Optimization Method

As introduced, the problem stated in “Model Context and Problem Definition” section has been addressed with a multimodal optimizer, i.e., an optimization algorithm designed to concurrently obtain multiple different global and local solutions to a problem (Sareni and Krähenbühl, 1998; Shir et al., 2010). It is the Universal Evolutionary Global Optimizer (UEGO) proposed by Jelasity et al. (2001).

As can be deduced from its name, UEGO is an evolutionary optimization algorithm (Lindfield and Penny, 2017), so it works with a population of solutions and simulates their Darwinian evolution to progressively achieve better solutions. However, it belongs to the memetic category of EAs (Moscato, 1989; Molina et al., 2011). This kind of method is characterized by promoting the autonomous behavior of candidate solutions as self-evolving agents in conjunction with the underlying evolutionary environment. Thus, in practical terms, a memetic method combines a generic evolutionary stage of global scope with a replaceable local search component. UEGO meets this requirement, which makes it highly adaptable to different optimization problems (Ortigosa et al., 2007; Redondo, 2009; Cruz et al., 2018).

**Figure 2:**
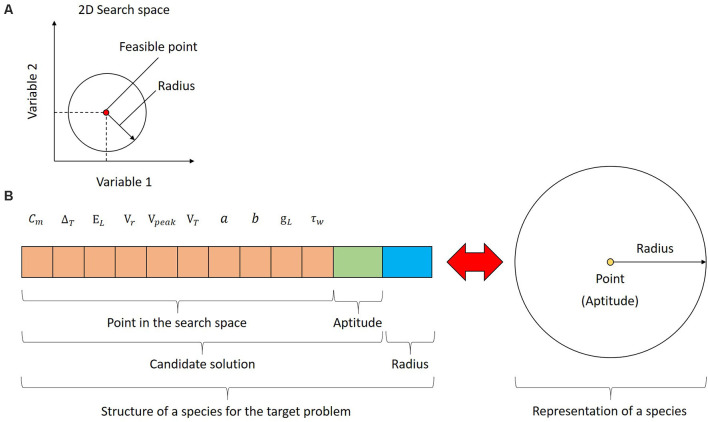
Species in UEGO. **(A)** Species in the search space for a hypothetical bi-dimensional problem. The candidate solution is a feasible point, and it is linked to a particular radius around it to define a region to center the search. Both define a circumference in a two-dimensional Euclidean space, but the concept can be extended to any dimensions through hyperspheres. **(B)** Structure of a species for the problem at hand (on the left) and its conceptual meaning (on the right). As shown on the left, in practical terms, a species consists of three parts: (1) a feasible point in the search space with a component for each optimization variable (colored in orange); (2) the fitness of the referred point according to the objective function (colored in green); and (3) the radius linked to this species (colored in blue). UEGO will use the referred information to build a hypersphere from every species, and they will be ultimately treated as exploration windows in the search space. This aspect is shown on the right side of the figure using a plain circumference due to the impossibility of showing the corresponding hypersphere.

The population of UEGO consists of different species, which is a fundamental concept for this method. Species are not plain candidate solutions as it occurs with standard GAs. Instead, every species combines a feasible and ranked candidate solution with an assigned radius around it in the search space. The radius is defined as a Euclidean distance to study the separation between different candidate solutions, i.e., to assess their similarity. Thus, a species defines a (hyper)sphere in the search space, and it is treated as an exploration window to center the independent local search component. Figure 2A depicts a sample species for a hypothetical optimization problem of two variables. As can be seen, the species represents both a candidate solution and a region in the search space on which the local search will focus. Since the referred example assumes two variables, the species can be easily visualized as circumferences. This is not the case for the problem at hand because the search space has 10 dimensions, i.e., the parameters to fit, and species will be hyperspheres in a 10-dimensional Euclidean space. Nonetheless, the underlying idea remains unaltered, and species will be generally depicted as circumferences for practical reasons. Figure 2B shows the structure of every species for the target problem and how UEGO perceives it.

As an algorithm, UEGO focuses on managing a population of different species, which defines its evolutionary part. It executes the steps shown in Algorithm 1. They are summarized next for the sake of self-completeness, but the interested reader is referred to Jelasity et al. (2001) and Ortigosa et al. (2001) for further details.

The algorithm takes the following parameters as input: (1) the maximum number of species to keep in the population (M); (2) the maximum number of evaluations of the objective function (N); (3) the minimum radius to keep between different species (r); and (4) the number of search levels or full cycles (l). After preliminary experimentation, these parameters have been set to *M* = 100, *N* = 10,000,000, *r* = 0.7, and *l* = 50 for the present study. The maximum number of species and function evaluations agree with the reference values proposed by Ortigosa et al. (2001), radii below 0.7 resulted in too many almost-equivalent solutions in this case, and the number of levels was progressively increased up to ensure that the search lasts enough, and the best-performing solutions are competitive.

**Figure 3:**
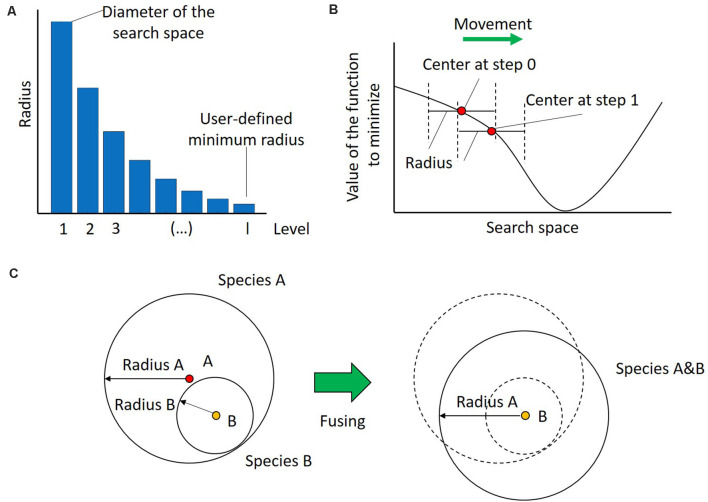
Dynamics in UEGO. **(A)** Evolution of the radius length linked to the species created at every level or cycle. The radius of the first level, i.e., that assigned to the initial species, is equal to the diameter of the search space. Subsequent radii decrease in geometrical progression until the last one, which corresponds to the minimum radius defined by the user to consider different solutions. By proceeding this way, species of the first cycles have more mobility, which promotes exploration of the search space, and those of the last levels have more exploitation, which helps convergence. **(B)** Movement of a species in a hypothetical single-dimension search space after an iteration of the local search method. The whole species is moved after finding a better center. Notice that the new point falls within the original region defined by the species, but its new position also moves the search focus. **(C)** Fusing species. On the left two overlapping species, A and B, with radii Radius A and Radius B, respectively. On the right, the species A&B that results from their fusion. This new species keeps the largest radius of both, i.e., Radius A, which aims to keep the regions to explore as broad as possible to avoid premature convergence. Additionally, take into account that since the center of Species A&B is that of B, it can be concluded that Species B has a better, i.e., lower value of the cost function. Finally, notice how the new species has slightly moved from the original zones, so it will be possible to explore a new zone of the search space.

Having gathered the input, the first step of UEGO is the initialization of its population. For this purpose, it randomly selects a point in the search space, evaluates it, and assigns the first radius to it. By definition, the radius of the first species is equal to the diameter of the search space, which is computed as the Euclidian distance between the lower and upper bounds. Therefore, the region defined by the initial species covers the whole search space, and no solution will be unreachable. After that, the radii assigned to species at creation, and hence the region that they cover, will decrease. They do in geometrical progression with the number of levels until the last one, which is linked to the minimum radius specified by the user (see Figure 3A). This strategy of progressively reducing the mobility at search is known as cooling in the field of Optimization, and it is inspired by the process of annealing metal (Lindfield and Penny, 2017). It promotes exploration at the beginning to find the best zones of the search space, avoids premature stagnation, and forces convergence at the end.

The second step in Algorithm 1 shows the memetic nature of UEGO. Namely, it consists of launching the local search component. This stage, also seen in step 8, independently affects every species in the population, but at this point there only exists the initial one. As introduced, local search is treated as an isolated component, and the method selected is briefly described at the end of this section. It is only required to start at the center of the species and find a better point in its region after several movements. Theoretically, the local search algorithm is limited by the region of the species, i.e., the radius linked to its starting point. Thus, no single step made by the optimizer in a given species can be larger than the radius. However, every time that the local search component finds a better point in the region, it becomes the new center of that species, so they are countinously moving in the search space. Figure 3B illustrates this concept. Aside from defining the initial point and the maximum step size for the local search method, UEGO also controls its computational budget, i.e., its number of function evaluations. The details about how UEGO distributes the total function evaluations allowed are out the scope of this work, but they are covered in depth in Jelasity et al. (2001) and Ortigosa et al. (2001). Regardless, the principle followed is to allow more function evaluations for later search levels, when it should be more interesting to explore the promising regions previously found. Also, notice that the local search algorithm should try to save function evaluations by early terminating when it finds itself unable to locate a better solution, so not all the allowed function evaluations might be consumed.

The two previous steps form the first level of search for UEGO. For this reason, the loop in Step 3 counts from 2. Notice that if the number of levels were set to 1 by the user, the algorithm would be mainly equivalent to launching the local search method from a random point. The result would hence be the single species after being locally optimized. Nonetheless, this situation is mostly theoretical. A standard configuration of UEGO is expected to execute several levels of search or cycles. Each of them consists of Steps 4–9.

Step 4 defines the computation of the radius that will be assigned to any new species created at the current level. As introduced, they decrease in geometrical progression. This step also involves determining the number of function evaluations that can be consumed for creating and locally optimizing species in Steps 5 and 8, respectively. The budget for creation is always three times the maximum number of species allowed, but that of local optimization increases with the number of levels as summarized above. See Jelasity et al. (2001) and Ortigosa et al. (2001) for further information.

Step 5 is where UEGO tries to increase its population. It first divides the creation budget among the existing species to calculate how many points will be allowed to evaluate. After that, within the region defined by every species, the algorithm randomly takes the permitted number of candidate solutions. Then, the points of every species are systematically paired with each other, and their middle points are evaluated. If the solution at any of the middle point is worse than that at its extremes, both members of the pair define new species. This is done under the assumption that they are on different sub-areas in the search space. The radius assigned to these new species will be the one that corresponds to the current level, which should be lower than any previous one. By proceeding this way, multiple new species will appear within the limits of every existing one and focusing on smaller regions to concentrate the search. Additionally, notice that UEGO will update the center of the initial species if any of the candidate points considered in their regions is a better solution, so they can move.

Step 6 scans the current population to check if the center of any pair of species is closer to each other than the radius of the current level. The goal is to avoid spending too much computing time separately exploring the same region. Species that overlap according to this criterion are fused into a single one. More specifically, the center of the resulting one is that which represents a better solution. The radius will be the largest one of them, which aims to keep the scope of search as broad as possible to avoid premature convergence. Figure 3C depicts this step assuming that species B is better than A, but it has a shorter radius. Notice that this definition ensures that there will always be a species whose radius covers the whole search space derived from the initial one, so it is always possible to reach any point in the search space.

Step 7 checks the length of the current population. If there are more species than allowed by the user through parameter M, those with the shortest radius are removed until the population size is in the valid range again. The removal criterion is aligned with the previous idea of maintaining species that allow escaping from low-performing local optima. The last two steps are both procedures already described. Namely, Step 8 will independently launch the local search component from every existing species, which will make them move around the search space. Step 9 rescans the population to identify and fuse any species that overlap after having been moved. The UEGO algorithm ends with step 11 by returning the surviving species. According to the process described, they are expected to be different promising solutions. The separation between them in the search space, i.e., degree of difference, will be the minimum radius defined by the user at least. Therefore, as intended, the users of this method and framework (Figure 1) have several options to study (final candidate selection) in case those solutions with the best numerical fitness do not appropriately meet the qualitative requirements that can be further analyzed in a subsequent stage.

Regarding the local search component previously referred to, the SASS or Solis and Wets’ method (Solis and Wets, 1981; Molina et al., 2011) has been used. It is a stochastic hill-climber that starts at the center of the given species and randomly decides a direction to move. The amplitude of every jump cannot exceed the radius of the species, and it is scaled depending on the number of positive (improving) and negative (non-improving) movements. This optimizer has been selected because it does not require any specific properties of the objective function. Besides, it has already been successfully used within UEGO (Ortigosa et al., 2007; Redondo, 2009). The configuration of this method is the recommended one. Namely, movements are made by adding a normally-distributed random perturbation vector with a standard deviation between 1e-5 and 1, starting at the upper bound and ultimately rescaled by the radius of the species. The standard deviation is doubled after five consecutive successful movements or halved after three consecutive failed ones. Notice that the local search method will terminate after 32 consecutive failed or discarded movements, no matter the remaining budget.

### Data Analysis

#### Multidimensional Scaling

To further illustrate the multimodal distribution of the different optimal solutions, we have applied the Classical Multidimensional Scaling (MDS) method (using scikit-learn Python library; Pedregosa et al., 2011). The distribution of the parameter values of the solutions through *n* dimensions (in our case, *n* = 10 parameters that define a neuron model, also named candidate solution) is denoted as landscape. Using MDS, the differences among landscapes were visualized as distances in the bi-dimensional plane. The input vector of distance to the MDS is calculated as a simple Euclidean distance between landscapes (as in other analysis works, such as in Rongala et al., 2018). Given a distance dot matrix, this algorithm recovers a representation of *D*-dimensional coordinates of data (in our case, *D* = 2 dimensions). This method allows studying the different landscapes chosen during the algorithm execution and represents their values in a 10-dimensional space embedded in a *2D* plot.

**Table:** 

Algorithm 1.- UEGO Algorithm	
*Input*: M, N, r, l	//Max. species, max. evaluation, min. radius, levels
1.- Initialize_List_Of_Species	//Create the first species
2.- Optimize_Species	//Launch the local search on it
3.- *for* i = 2 to l *do*:	//Main loop (Steps 1 and 2 are define the first level)
4.- Compute_Level_Config	//Manage the use of function evaluations and radii
5.- Create_Species	//Create species in the zones of the exising ones
6.- Fuse_Species	//Avoid that species overlap each other at this level
7.- Shorten_Species_List	//Remove species if there are more than allowed
8.- Optimize_Species	//Launch the local search on every existing species
9.- Fuse_Species	//Avoid that species overlap each other at this level
10.- *end for*
11.- Return_Surviving_Species	//The remaining species become the set of results

#### Technical Details for Reproducibility

Python (version 2.7.12) and MATLAB (version 2018b) implementations were used to launch the second stage of the workflow (the exploration processes of the multimodal algorithm). The proposed pipeline allows simulating the neuron models and calculating the features scores through the Python-NEST environment (Python Software Foundation Python 2.7.12, 2016; van Rossum, 1995) and NEST simulator 2.14.0 (Peyser et al., 2017) and evaluating and exploring different candidate solutions in optimization cycles through Python-MATLAB implementations. After considering 10 independent executions with different seeds, UEGO executes 50,000 function evaluations on average. The one selected for further analysis in “Results” section used 47,951, which approximately results in 32 h of run time in the execution platform. In the last stage of the workflow, the reproduction and validation of the resulting neuron models (candidate solutions) were analyzed using Python-NEST scripts. The Figures were generated using Matplotlib (version 2.2.5; Hunter, 2007; Caswell et al., 2020) and Numpy (version 1.16.6; Harris et al., 2020) libraries. The simulations were run with an Intel Core i7–4790 processor with 4 cores and 32 Gb of RAM. The source code and data are available in this public repository: https://github.com/MilagrosMarin/Multimodal-optimization-for-fitting-cerebellar-GrCs.

## Results

### Analytical Results

The results achieved by the optimization stage consist of a population of up to 100 candidate solutions, which is a user-given parameter. More accurately, the multimodal optimizer tries to find different yet promising parameter sets in the search space, and it uses a minimum user-given separation radius for this purpose (see the “Materials and Methods” section). For this purpose, through the search, the optimizer manages a population of feasible configurations that are distributed over the search space, can produce new ones, move, and absorb others when they are considered to represent the same parameter set. Thus, the number of candidate solutions that ultimately survive as results for further consideration by an expert might vary. Since the optimizer is stochastic, its results might vary between different executions, so it has been launched 10 times as mentioned in “Technical Details for Reproducibility” section. Supplementary Figure 1 shows that the results are similar between executions in terms of search space coverage and overall numerical quality. The one selected for further analysis in this section resulted in a final population of 25 different candidate solutions.

**Figure 4:**
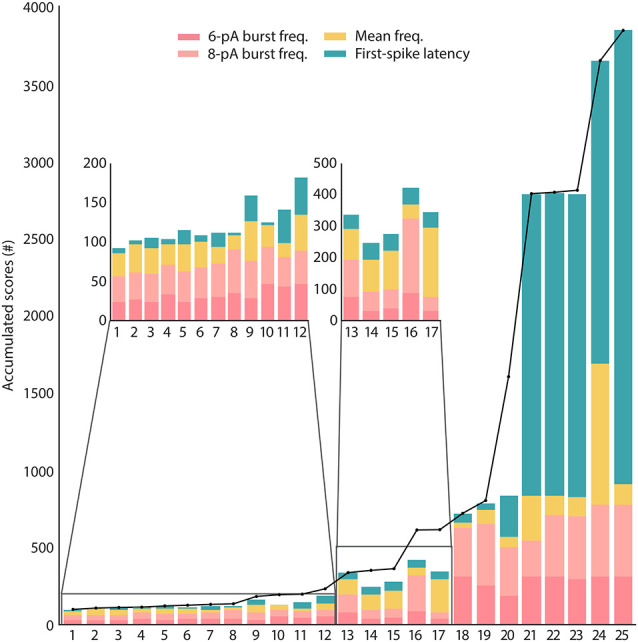
Scores of the candidate solutions. The total scores obtained from minimizing the features of the candidates according to the objective function are represented in black dots joined together by a black line. These total scores are unwrapped by the scores of each single feature (feature scores) for every candidate solution and represented in stacked bars. The feature calculations are explained in “Model Context and Problem Definition” section. The feature of burst frequency (pink bar plots) has been divided based on the sinusoidal stimulation amplitude of 6 pA or 8 pA (the standard deviation has not been included in the stacked bar).

These candidate solutions are minimized to the target multi-feature objective function and their scores are represented in Figure 4. The candidate solutions show different combinations of feature adjustments in order to reach the minimal total score, which reveals a well-balanced definition of the multi-feature objective function. Not unexpectedly, the spiking resonance feature contributed the most to the score (pink bars in Figure 4; as this is the feature with the highest number of *in vitro* reference points). Although they were selected by their well-ranked solutions, some of them (from 18 to 25) have low-performing configurations for some of the considered properties (mostly for the feature of latency to the first spike, green bars in Figure 4).

**Figure 5:**
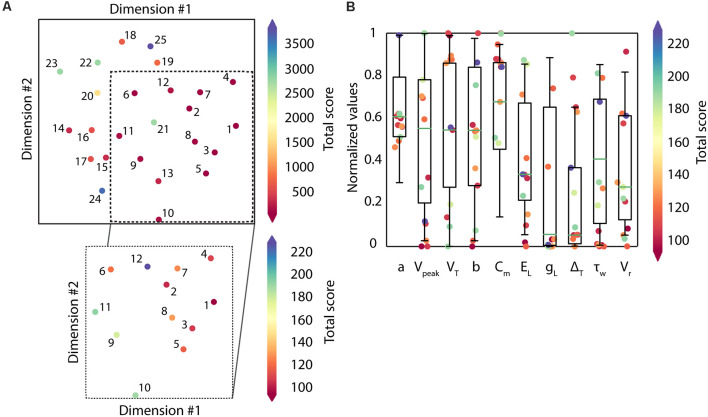
Distribution of the candidate solutions and their respective parameter values. **(A)** Representation of the population of solutions (*n* = 25) using Classical Multidimensional Scaling (MDS). The colormap shows the total score of each candidate solution, red-colored being the solutions with the lowest scores (best solutions). In zoom, the most representative candidates (those with scores under 250 units; *n* = 12). **(B)** Parameter distribution of the candidate solutions. Each dot corresponds to the parameter value that defines every candidate solution with the lowest scores (those with scores under 250 units; *n* = 12). The colormap reflects the total score obtained by each parameter. The boxes correspond to the interquartile ranges (IQR) between the first (Q1) and the third (Q3) quartiles. The green line represents the median among the values obtained for each parameter. The whiskers correspond to the 5- and 95- percentile respectively.

The different solutions in the search space expected to be returned by the algorithm are visualized using the MDS algorithm, evidencing the multimodality of the search space (Figure 5A). The candidate solutions with the lowest scores (under 250 units, which are the solutions from 1 to 12 and in warmer reddish colors) correspond to different high-quality solutions. They are sparsely located along the 2-dimensional display (zoom of the most representative candidates, and colored based on the total score of each candidate solution, in Figure 5A). The parameter configurations that define each of these solutions explored a variety of values from within their boundaries (e.g., *V_reset_, E_L_, τ_w_*), which means that the algorithm successfully went over the parameter space (i.e., landscapes; Figure 5B).

### Reproduction of Spiking Dynamics

In the section above, the candidate solutions of most interest (from 1 to 12) showed quantitative fitting to the supra-threshold characteristics defined in the multi-feature objective function with minimized scores. In this section, the top-ranked solutions are qualitatively analyzed regarding their accuracy in capturing this intrinsic excitability of cerebellar GrCs, i.e., firing discharge with a mean frequency increased whereas latency to the first spike firing decreased under injected currents and spiking resonance in the theta range under sinusoidal currents.

Although the top-ranked solutions achieved lower score values, this fact might not imply reproducing the complex firing dynamics of the neuron. The case of the spiking resonance is an appropriate example of this possibility: although the experimental points are suitably adjusted to the graphic curve, the neuron responses are larger when the neuron behavior is extrapolated to higher sinusoidal frequencies. That is, as mentioned in “Feature Measurement” in “Materials and Methods” section, the burst frequencies generated with stimulation frequencies beyond 10 Hz fell to zero because the *in vitro* measurements contained either one or no spikes. Since there are no points of burst frequencies in higher frequencies in the biological measurements, we have avoided including them in the objective function.

**Figure 6:**
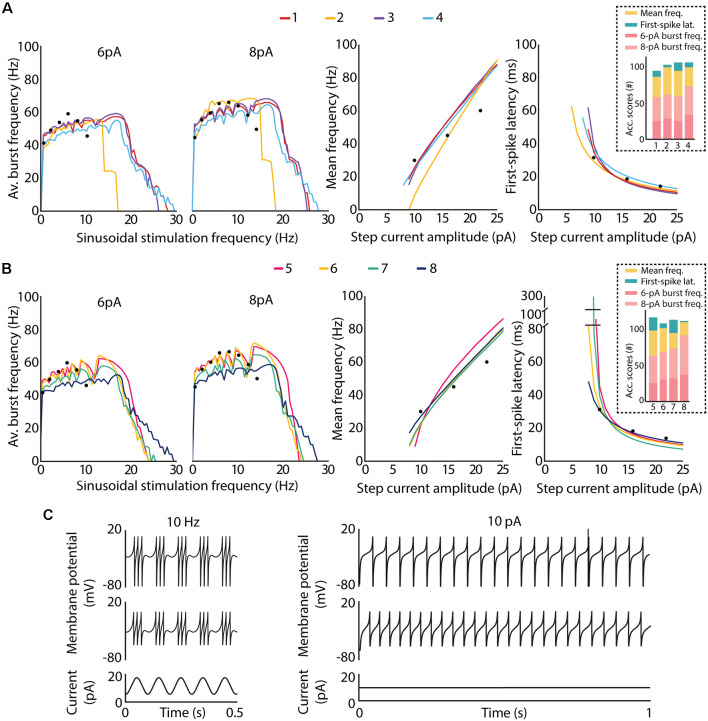
Intrinsic excitability of the top-ranked neuron models of cerebellar GrCs. The optimal candidate solutions are qualitatively analyzed according to the features defined in the objective function, i.e., spiking resonance in the theta range under sinusoidal stimulation (left plots), mean frequency (middle plots) and latency to the first spike (right plots), the last two under direct current stimulation. Black dots represent the experimental data used as reference in the optimization process. **(A)** Spiking dynamics of the top-four candidate solutions of the final population. Their accumulated scores of each feature are represented as bars in the dashed box. **(B)** Spiking dynamics of the candidate solutions ranked 5–8 of the final population. Their accumulated scores for each feature are represented as bars in the dashed box. **(C)** Example traces from two of the best candidate solutions (top plots from candidate 5 and middle plots from candidate 6) show: on the left, spike bursts from both neurons under sinusoidal current injection of 10-Hz frequency, 6-pA amplitude and 12-pA offset, as it is represented in the bottom plot; on the right, repetitive spike discharge from both neurons during a step-current injection of 10 pA, which is represented in the bottom plot.

The whole subset of interesting candidates (from 1 to 12) reproduced the complex spiking behaviors within the limits of our electrophysiological observations. The parameter values obtained for the set of solutions are contained in Table 2. Candidates 1–8 manage to reproduce all the three spiking features mentioned above (Figure 6). The spiking resonance curves (left plots in Figure 6) were successfully replicated (with preferred frequencies around 6–20 Hz) by the whole subset of candidates. The intensity-frequency (I-F) plots (middle plots in Figure 6) were almost linear between 0 and 100 Hz and the latencies to the first spike were also replicated (right plots in Figure 6) as in the biological piece of evidence (D’Angelo et al., 2001).

**Table 2:** Parameter configurations of the best-ranked candidate solutions and their total score.

Candidate solution	Parameter configuration										
	*a (nS)*	*V_peak_ (mV)*	*V_T_ (mV)*	*b (pA)*	*C_m_ (pF)*	*E_L_ (mV)*	*g_L_ (nS)*	* Δ_T_ (mV)*	*τ_w_ (ms)*	*V_r_ (mV)*	Total score
1	0.123	−19.981	−20.446	−0.999	4.226	−79.225	0.333	55.881	7.138	−76.638	93.992
2	0.202	−7.078	−38.149	0.140	4.400	−67.194	0.001	54.382	73.441	−43.458	102.906
3	0.223	−19.984	−54.527	−0.429	4.408	−66.527	0.003	653.468	1.700	−71.568	106.522
4	0.139	−15.731	−20.000	1.000	4.998	−76.037	0.001	792.296	12.717	−56.917	108.708
5	0.244	7.841	−24.830	−0.946	4.741	−79.985	7.413	55.820	1.039	−79.972	116.652
6	−0.010	3.859	−24.929	0.081	4.324	−63.421	3.742	36.480	790.811	−55.069	121.169
7	−0.069	−18.914	−24.208	0.089	4.303	−51.501	0.342	1.116	273.243	−77.872	126.610
8	0.126	8.171	−25.613	−0.267	3.423	−73.981	0.005	631.309	8.875	−67.973	130.303

With respect to the qualitative adjustment of the best-ranked solutions, the top-four candidates reproduce all the three spiking behaviors. More specifically, candidate 2 reproduces the spiking behaviors according to experimental registers of real cells (Figure 6A). Other best-ranked candidates, such as 1, 3 and 4, also reproduce qualitatively these behaviors as the reference reports, but with resonance curves (around 5–20 Hz, as seen in left plots in Figure 6A) slightly shifted out of the concrete theta band of *in vitro* cerebellar GrCs (around 6–12 Hz in D’Angelo et al., 2001).

Regarding the quantitative comparison of these best-ranked solutions (the distance of the feature values from the experimental measurements defined in the integrated objective function), candidate 2 obtained the highest score for the concrete points of the mean frequency feature (yellow bar in the dashed box in Figure 6A), but the lowest score for the first-spike latency feature (green bar in the dashed box in Figure 6A). That is, candidates 1, 3 and 4 obtained lower scores for the mean frequency feature than candidate 2.

This fact together with the shifted resonant curves in higher preferred frequencies may indicate an incompatibility of both firing properties (i.e., the repetitive spike discharge and the spiking resonance), within the AdEx models, as we previously hypothesized in Marín et al. (2020). Thus, the GrC behavior complexity in reproducing these features, being beyond the capabilities of these AdEx models with a single parameter configuration (GrCs have different functioning modes).

Similarly, candidates 5, 6, 7 and 8 reproduce all the features but with the resonance curves in slightly higher preferred frequencies (around 6–15 Hz; left plot in Figure 6B). In addition, candidates 5, 6 and 7 showed larger initial latencies (around 100, 80 and 300 ms, respectively) than the candidates mentioned before (i.e., 1,2,3,4 and 8, with initial latencies around 50–60 ms) which are closer to the experimental recordings used as reference (right plots of Figure 6B). Example traces generated from two of the best candidate solutions are shown in Figure 6C. In particular, the left plots of Figure 6C show the generation of spike bursts clustered in triplets or longer bursts in time slots corresponding to the positive phase of the sinusoidal current, as described in the reference report (D’Angelo et al., 2001). It is worth mentioning that the ISI during the burst duration (i.e., the oscillatory burst frequency defined in the fitness function) is very similar to that from real traces. In addition, the repetitive spike discharge, experimentally evidenced in cerebellar GrCs (D’Angelo et al., 1995, 2001), generated by these candidates are shown in the right plots of Figure 6C.

## Discussion

The present study illustrates the application of a novel optimization framework to the case of the cerebellar GrCs: the automated identification of different and promising configurations of the neuron model parameters to reproduce complex spiking behavior through multimodal algorithms for an expert to decide. The solutions produced by the multimodal optimization process represent a valuable analysis tool that facilitates better understanding of how certain neural model properties are supported by a specific parameter configuration. Two challenges were addressed: (1) the optimization of efficient neuron models that allow the replication of complex dynamics such as the spiking resonance in the theta frequency band while maintaining other typical GrC dynamics such as the regular repetitive firing and the spike timing; and (2) the generation of a diverse population of neuron models with widely explored configurations in sparse local minima. These challenges were addressed by optimizing a multi-feature fitness function defined with the distinctive characteristics of cerebellar GrCs. In this case, the spiking resonance in the theta-frequency band of the GrCs is a complex behavior believed to improve the information processing in the cerebellum (D’Angelo et al., 2001, 2009; Gandolfi et al., 2013). The mean frequency of repetitive firing and the spike timing (latency to the first spike) are the main properties of the GrC used to measure their intrinsic excitability. Addressing this approach through the optimization workflow resulted in the full-fledged exploration of a population of efficient neuron models that sufficiently reproduce highly realistic dynamics. Finally, it is also important to take into consideration a validation of the neuronal firing dynamics in order to analyze in detail the behavior of the obtained neuron models and how the parameter diversity can be steered to adapt the model to specific purposes or studies. The selected neuron models are presented as efficient tools that can formulate biological network hypotheses and shed some light on future neuroscientific research.

To solve neuron model tuning problems, it is possible to opt for methods based on robust mathematical principles whenever the objective function has some properties, such as being expressed by a particular type of analytical formulation and being differentiable. For instance, the point-neuron model of a cerebellar GoC proposed by Geminiani et al. (2018) was modified in order to optimize part of its parameters using a SQP algorithm from spike voltage traces under input current steps. The SQP algorithm uses differential calculus in locating the optimum points and allows to simultaneously minimize the objective function and the constraint function. An alternative to these methods are the EAs, such as GAs, and the PSO, which allow solving parameters tuning problems that classical methods might fail for multidimensional non-linear systems, such as the AdEx model. These algorithms provide high flexibility, universality (being able to be applied to different cases) and proved to be fast and efficient strategies to take into consideration for fitting neuron models (Cachón and Vázquez, 2015; Van Geit et al., 2016; Shan et al., 2017). This is the case of the optimization of an AdEx model of a cerebellar granule cell (GrC) and a Golgi cell (GoC) proposed in Nair et al. (2015). The fitness function measured the similarity between spike trains from spiking traces. However, the PSO algorithm was modified since all the solutions of the search did not result in a feasible solution due to the non-linear dynamics of the AdEx equations. In our previous study (Marín et al., 2020), we optimized an AdEx neuron model of a cerebellar GrC based on specific features (not whole traces) from *in vitro* recordings using “simple GA”. In Marín et al. (2020), we proposed a single final candidate solution as the best approximation of the multi-feature fitness function of the cerebellar GrCs. However, in this work we take a step further in finding and fitting multiple neuron model configurations in a single run based on such a complex fitness function. This allows a detailed analysis of how neuron properties are supported by specific parameter configurations.

**Figure 7:**
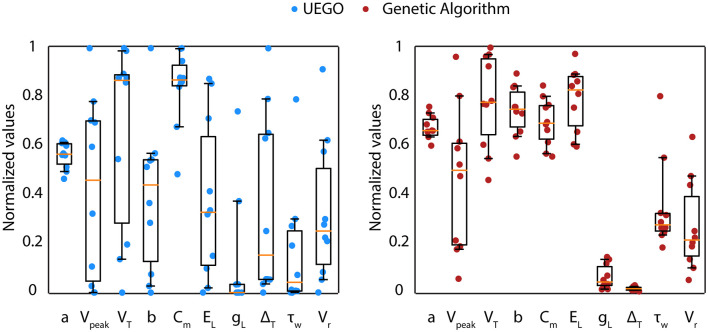
Parameter distribution of candidate solutions from UEGO and GA. Comparison between the parameter values obtained from the best-ranked candidate solutions (from 1 to 10) in the execution of UEGO (left—blue circles) and from the best single solution of 10 independent executions of the GA (right—red circles). Each dot corresponds to the parameter value that defines every candidate solution. The boxes correspond to the IQR between the first (Q1) and the third (Q3) quartiles. The orange line represents the median among the values obtained for each parameter. The whiskers correspond to the 5- and 95- percentile respectively.

The objective of the present study is not to promote UEGO as the most effective algorithm in plain values of the objective function. Instead, the aim is to define an alternative framework that relies on this multimodal method for gathering and studying heterogeneous model configurations with independence of strictly being the best ranked. However, notice that UEGO can numerically compete with the results achieved by the GA used in the reference work (Marín et al., 2020). For the sake of completeness, the mean results of the GA proposed in Marín et al. (2020), which was the initial option for solving the problem at hand, have been compared to the mean results of the best-ranked solutions of the UEGO execution described in this article (Table 3). The referred GA took 30,000 function evaluations, but UEGO executes 50,000 on average with the configuration proposed, which is almost twice. For this reason, the number of cycles of the genetic method has been doubled to increase its exploration possibilities and take comparable computational effort. While the GA allows obtaining a unique best solution (low score), a multimodal algorithm such as UEGO allows generating multiple candidate solutions that reproduce reasonably well the neuron behaviors with wider parameter configurations (Figure 7).

**Table 3:** Comparative table of best solutions from UEGO and regular GA.

Method	a (nS)	V_peak_ (mV)	V_T_ (mV)	b (pA)	
UEGO	0.12 ± 0.10	−3.06 ± 14.25	−34.48 ± 14.67	−0.22 ± 0.59
GA	0.34 ± 0.09	−1.69 ± 11.24	−28.96 ± 7.23	0.48 ± 0.20
	C_m_ (pF)	E_L_ (mV)	g_L_ (nS)	Δ_T_ (mV)
UEGO	4.23 ± 0.72	−64.87 ± 12.61	1.18 ± 2.35	353.29 ± 357.56
GA	3.49 ± 0.45	−48.59 ± 4.95	0.63 ± 0.48	15.53 ± 7.33
	τ_w_ (ms)	V_r_ (mV)	Total score
UEGO	166.41 ± 236.07	−66.93 ± 11.04	127.43 ± 30.13
GA	346.13 ± 177.24	−69.26 ± 7.06	107.61 ± 5.04

*The table shows the mean values and standard deviations of each neuron parameter from the best candidate solutions of UEGO (candidate solutions from 1 to 10) and each of the single best solutions from 10 independent executions of regular GA (with different seeds). Note that the dispersion of the parameters is considerably wider in UEGO*.

Our aim is to provide a set of feasible, promising, and well-distributed solutions that result from numerical optimization for an expert to select the most appropriate one. The multimodal algorithms allow adjusting the exploration and consequent extraction of more than one candidate solution through the parameter landscapes (understood as the “space” of possible parameter values that a solution can take after the optimization process). An advantage of using a multimodal optimization method is that it results in a population of candidate solutions which is diverse in terms of parameters values as they best fit the target features at different areas in the parameter space. This allows using the candidate solutions as the substrate for a detailed parameter analysis with respect to the neuron model desired properties. In addition, the algorithm can adjust the parameter exploration according to different working ranges (wide exploration within the parameter boundaries). This population of final candidate solutions characterizes the behavior of the neuronal dynamics across the parameter space, i.e., how neuronal dynamics change as the parameters are modified (they can complement or conflict with each other towards optimizing a multi-feature fitness function, finding trade-offs among parameters in a solution). The expert is able to match the best parameter set ups towards optimizing one specific feature or another, or rather select a parameter set up to fit all the different target features at the same time (avoiding one feature to dominate against other ones within the combined cost function). The exploration and extraction of a diverse population of solutions facilitate the analysis process of how specific parameters ranges help to adjust particular features (the algorithm might perform an unbalanced adjustment of features, focusing more on some of them and distances to others). The multimodal optimization, in spite of being a more specific and robust engine (and so, more laborious), implies a suitable alternative for detailed exploration and analysis of the neuronal dynamics against the single candidate solution obtained by other simpler algorithms which might lose biological information necessary for the subsequent study.

### Future Implications

The novel workflow presented here constitutes a flexible and versatile tool that can be generally applied to this level of complexity with other commonly used point-neuron models, such as the GLIF or integrate-and-fire neuron models, and with other types of spiking dynamics, as long as the electrophysiological data is available. The multimodal optimization algorithm is only led by the value of the objective function, but this approach does not determine the goodness of the solution (the minimized score), although it is capable of exploring a biodiverse population of solutions according to pre-optimized solutions with interrelated parameters. The pre-optimization allows filtering the solutions according to numerically promising configurations. This facilitates the analysis of the parameter space in relation to the desired neuron properties. The post-optimization is based on the decision of the user. This proposal is an automatization of the population diversity of plausible neuron models for complex spiking behaviors.

In our results we already have seen certain biodiversity in the parameter configurations of the final population that can lead to a specific behavior shown by biological cells. If the heterogeneity of GrCs is a real fact in the biology of granule cells, then it could be also reflected in the variability of neuronal dynamics of the neuron models that reproduce the same target behavior (Lengler et al., 2013; Migliore et al., 2018). Regarding the biological data used as reference, in this article we generate a heterogeneous population of neurons based on different parameter configurations of the AdEx model and mimicking the neuronal behavior extracted from biological data. In future work, it would be of outstanding interest to optimize from a population of real cerebellar neurons that show variations in the target behaviors so that diversity can be explicitly captured. The construction of a multi-objective fitness function, compounded by several error functions that all have to be optimized simultaneously, could be a future extension of the presented workflow in order to analyze the Pareto front of all the possible parameter configurations. This would allow exploring the direct relationships among parameters and single features.

### Concluding Remarks

In this article, we present a novel and robust optimization framework integrating a multimodal algorithm that co-optimizes the spiking resonance in the theta-frequency band, the repetitive spiking discharge and the latency to the first spike in efficient models of cerebellar GrCs. The validity of the framework is confirmed by analyzing the electrophysiological predictions of the biological characteristics. The proposed methodology will be reflected as ease-of-use through the following workflow, even though a multimodal algorithm usually requires high knowledge of the field and it is difficult to use for non-expert users. The UEGO algorithm exhibits its strength in adapting to the complex data structure associated with the neuron dynamics. The optimization workflow helps to easily generate a population of functional neuron models. In addition, employing a multimodal algorithm plays a key role in the proposed workflow to help the exploration of different local minima. The outcomes of the optimization study show promising results that successfully establish the solution repository considering multiple features in the function. Such results are verified by presenting the spiking resonance, repetitive firing and timing curves and the dominated solutions. According to the analytical results, the candidate solutions exhibit a consonant relationship between the features, meaning that the algorithm does not need to make a decision to balance the trade-off benefits (equilibrated distributions). The efficient models and features obtained in this work are mainly to demonstrate the feasibility of the proposed optimization workflow. It can be easily modified by other types of point-neuron models (such as GLIF) or other neuron characteristics in future work. The application to the case of cerebellar GrCs implies taking a step further towards advanced exploration of candidate solutions. It facilitates the evaluation of models based on different neuronal parameters which represent various internal neuronal mechanisms to achieve the target spiking behaviors defined in a complex fitness function.

## Data Availability Statement

The methodology and datasets presented in this study can be found in an online repository. The repository can be found at: https://github.com/MilagrosMarin/Multimodal-optimization-for-fitting-cerebellar-GrCs.

## Author Contributions

MM and NC: study design. MM, MS-L, and EO: literature and database search. NC and EO: methodology. MM, JG, MS-L, and RC: analysis and interpretation of results. MM, NC, and JG: writing of the article. All the results included in this article are part of MM’s PhD thesis. All authors contributed to the article and approved the submitted version.

## Conflict of Interest

The authors declare that the research was conducted in the absence of any commercial or financial relationships that could be construed as a potential conflict of interest.

## Abbreviations

AdEx: Adaptive exponential integrate-and-fire
EA: Evolutionary algorithm
GLIF: Generalized leaky integrate-and-fire
GA: Genetic algorithm
GoC: Golgi cell
GrC: Granule cell
HH: Hodgkin-Huxley
PSO: Particle swarm optimization
I-F: Intensity-frequency.
