# Supplementary figures and images for: On the Use of a Multimodal Optimizer for Fitting Neuron Models. Application to the Cerebellar Granule Cell
Source: Front Neuroinform. 2021 Jun 3;15:663797. doi: 10.3389/fninf.2021.663797 (PMC8209370; doi:10.3389/fninf.2021.663797)

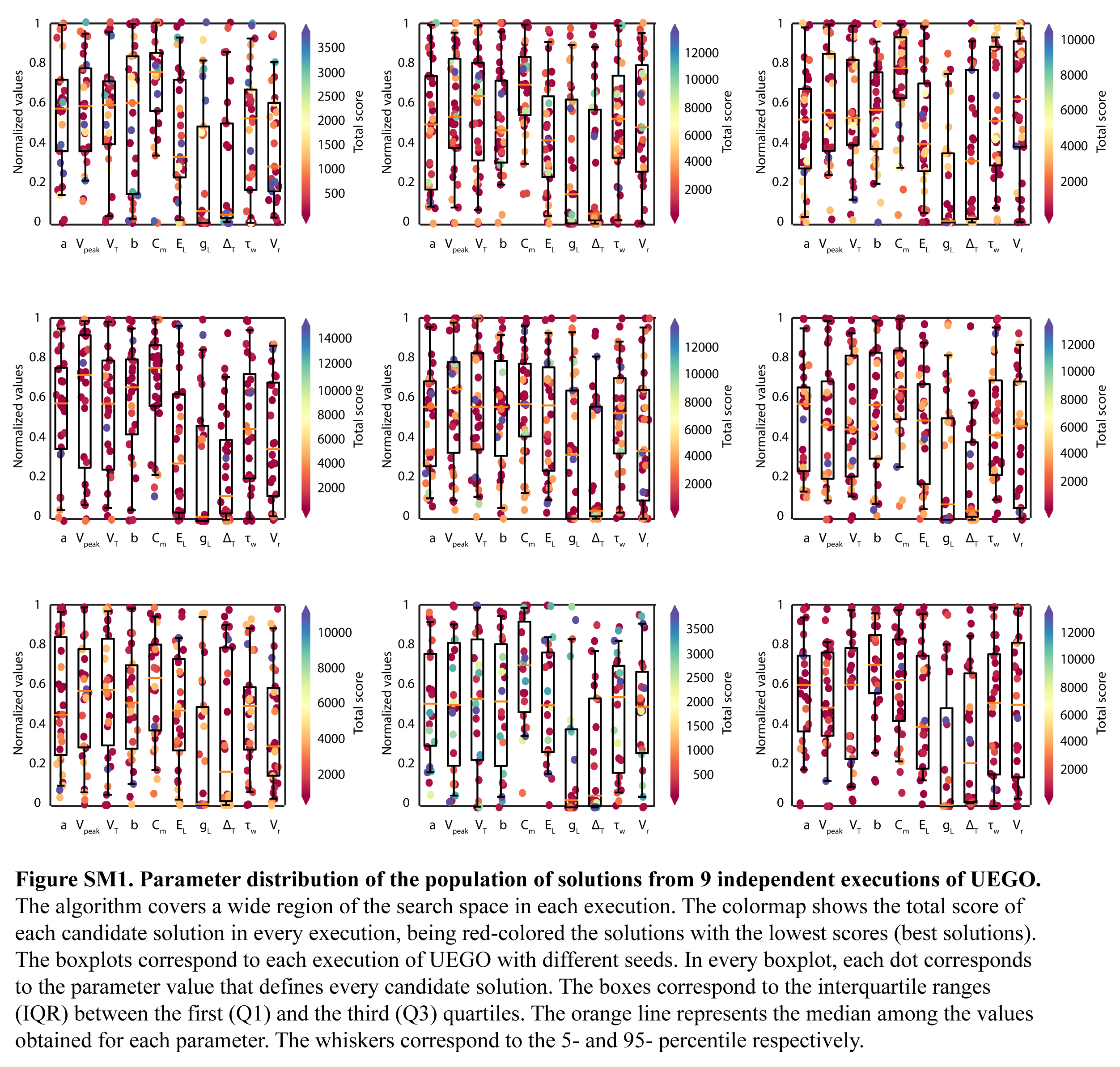

Supplement: Supplementary file 1 [file Image_1.TIF]
